# Supplementary material for: Resolving the full spectrum of human genome variation using Linked-Reads
Source: Genome Res. 2019 Apr;29(4):635–45. doi: 10.1101/gr.234443.118 (PMC6442396; doi:10.1101/gr.234443.118)
Supplement: Supplemental Material [file supp_gr.234443.118_Supplemental_longranger-2.2.zip › longranger-2.2.tar.bz2!/lr2.2-public/lib/stan/cmdstan/stan/doc/api/html/classstan_1_1mcmc_1_1base__leapfrog-members.html]

Stan: Member List


|  |  |
| --- | --- |
|  | Stan  2.5.0  probability, sampling & optimization |


- Main Page
- Related Pages
- Namespaces
- Classes
- Files

- Class List
- Class Index
- Class Hierarchy
- Class Members

All Classes Namespaces Files Functions Variables Typedefs Enumerations Enumerator Friends Macros Pages

- stan
- mcmc
- base\_leapfrog

stan::mcmc::base\_leapfrog< H, P > Member List

This is the complete list of members for stan::mcmc::base\_leapfrog< H, P >, including all inherited members.

|  |  |  |
| --- | --- | --- |
| base\_integrator(std::ostream \*o) | stan::mcmc::base\_integrator< H, P > | inline |
| base\_leapfrog(std::ostream \*o) | stan::mcmc::base\_leapfrog< H, P > | inline |
| begin\_update\_p(P &z, H &hamiltonian, double epsilon)=0 | stan::mcmc::base\_leapfrog< H, P > | pure virtual |
| end\_update\_p(P &z, H &hamiltonian, double epsilon)=0 | stan::mcmc::base\_leapfrog< H, P > | pure virtual |
| evolve(P &z, H &hamiltonian, const double epsilon) | stan::mcmc::base\_leapfrog< H, P > | inlinevirtual |
| out\_stream\_ | stan::mcmc::base\_integrator< H, P > | protected |
| update\_q(P &z, H &hamiltonian, double epsilon)=0 | stan::mcmc::base\_leapfrog< H, P > | pure virtual |
| verbose\_evolve(P &z, H &hamiltonian, const double epsilon) | stan::mcmc::base\_leapfrog< H, P > | inline |


---

[ Stan Home Page ]

*© 2011–2014,
Stan Development Team.*
